# A Novel Mutation of Transferrin Receptor 2 in a Chinese Pedigree With Type 3 Hemochromatosis: A Case Report

**DOI:** 10.3389/fgene.2022.836431

**Published:** 2022-04-08

**Authors:** Shan Tang, Li Bai, Yuan Gao, Wei Hou, Wenyan Song, Hui Liu, Zhongjie Hu, Zhongping Duan, Liaoyun Zhang, Sujun Zheng

**Affiliations:** ^1^ Beijing Youan Hospital, Capital Medical University, Beijing, China; ^2^ The First Hospital of Shanxi Medical University, Taiyuan, China

**Keywords:** case report, TFR2 gene, compound heterozygous mutation, hereditary hemochromatosis, Chinese

## Abstract

Type 3 hereditary hemochromatosis (HH) is a rare form of HH characterized by genetic mutation in the *TFR2* gene. Clinical features reported in patients with type 3 HH include abnormal liver function, liver fibrosis, cirrhosis, diabetes, hypogonadism, cardiomyopathy, and skin pigmentation. Since its original description in 2000, 33 pathogenic *TFR2* mutations associated with HH have been described until now. Here, we first reported a Chinese pedigree of *TFR2*-related hemochromatosis with a novel compound heterozygous mutation c.1288G > A (p.G430R)/c.960T > A (p.Y320X). Interestingly, different phenotypes were reported although the proband and his sister shared the same gene mutation. This inconsistency between genotypes and phenotypes indicates multifactorial etiology contributing to the development of HH. Our report broadens the mutation spectrum of the *TFR2* gene associated with HH.

## Introduction

Hereditary hemochromatosis (HH) is defined as an inherited iron overload disorder characterized by excessive absorption of iron (Kowdley et al., 2019). Over time, iron deposits in multiple organs including the liver, pancreas, heart, joints, and pituitary gland, further leading to liver cirrhosis, restrictive cardiomyopathy, heart failure, arthropathy, skin pigmentation, diabetes mellitus, and so on (Bacon et al., 2011). HH can be classified as types 1, 2, 3, and 4. Type 1 HH caused by the *HFE* gene mutation is the most prevalent form of HH in Caucasians (Brissot et al., 2018). Mutations in hemojuvelin BMP co-receptor (*HJV*) and hepcidin antimicrobial peptide (*HAMP*) genes (type 2a and 2b HH, respectively) result in juvenile hemochromatosis, the most severe form of HH (Papanikolaou et al., 2004). Type 4 HH, also known as ferroportin (FPN) disease, is the only autosomal dominant form of hemochromatosis due to the mutations in the solute carrier family 40-member 1(*SLC40A1*) gene (Joshi et al., 2015). Type 3 HH is a rare form of HH characterized by the genetic alterations in the transferrin receptor 2 (*TFR2*) gene, with an estimated allele prevalence between 0.0001 and 0.0004 among European populations (Wallace and Subramaniam, 2016). The human *TFR2* gene is located on chromosome 7q22 and encodes transferrin receptor 2. TFR2 is a type II transmembrane glycoprotein, a member of the TFR family. It provides iron to the cell by internalization of the transferrin iron complex through receptor-mediated endocytosis (Silvestri et al., 2014). Hepcidin is the central regulator of systemic iron homeostasis, which can not only prevent absorption of iron from the gut but also prevent the release of iron from macrophages (Worthen and Enns, 2014). Hepcidin expression at an abnormally low levels leads to iron overload. Hemochromatosis, which is associated with mutations in *TFR2,* usually occurs at an earlier age than HFE-related hemochromatosis and usually has a more severe phenotype (Pietrangelo, 2010).

Here, we described a Chinese pedigree presented with typical features of HH. Also, we found a novel compound heterozygous mutation in the *TFR2* gene, namely, c.1288G > A (p.G430R)/c.960T > A (p.Y320X). To the best of our knowledge, this is the first report on a Chinese pedigree of *TFR2*-related hemochromatosis.

## Case Presentation

The patient (Figure 4, II-1), a 48-year-old male, was admitted to Beijing YouAn Hospital due to liver cirrhosis. Abdominal ultrasound suggested cirrhosis and splenomegaly 4 months ago even when the patient had no discomfort. In another hospital, he was diagnosed with diabetes and treated with insulin. He hardly ever drank and denied a family history of liver diseases. Physical examination revealed that there are no other abnormalities except for skin pigmentation.

A thorough examination was carried out to find out the cause of cirrhosis. The liver function test showed that the levels of alanine aminotransferase (ALT) and aspartate aminotransferase (AST) were elevated (Table 1). Serum markers for viral hepatitis and autoimmune liver diseases were all negative. Notably, the patient had abnormal plasma iron indices: iron, 47 μmol/L; transferrin saturation, 97.9%; and ferritin>2000 ng/ml. HbA1c was significantly elevated. Testosterone was normal (18.26 nmol/L).

**TABLE 1:** Laboratory test results of the proband and the proband’s family.

	Proband (II-1)	Proband’s sister (II-3)	Proband (II-1)	Proband’s sister (II-3)	Normal range
			1 year later	1 year later	
Alanine aminotransferase (U/L)	113	19	39	30.7	7–40
Aspartate aminotransferase (U/L)	84	23	56	32.23	13–35
Total bilirubin (μmol/L)	21	6.3	14.3	9.5	5–21
Direct bilirubin (μmol/L)	5.1	1.6	4.3	3.1	0–7
Total bile acid (μmol/L)	52.2	9.8	15.7	—	0–10
Alkaline phosphatase (U/L)	112	116	150	108.1	50–135
γ-glutamyltransferase (U/L)	35	11	25	15.8	7–45
Albumin (g/L)	44.7	41.3	45.7	43.7	40–55
White blood cells (*10^9/L)	8.63	5.89	6.51	5.76	3.5–9.5
Red blood cells (*10^12/L)	5.04	4.18	4.52	4.86	4.3–5.8
Hemoglobin (g/L)	157	137	144	156	130–175
Platelets (*10^9/L)	157	219	136	123	125–350
Hepatitis B virus surface antigen	Negative	Negative	—	—	Negative
Anti-hepatitis C virus	Negative	Negative	—	—	—
Iron (µmol/L)	47	37	48	37.1	<25
Transferrin saturation (%)	97.9	94.9	77.5	83.6	<45%
Ferritin (ng/ml)	>2000	709	>2000	545.8	13–150
Total iron-binding capacity (μmol/L)	48	39	62	44	45–75
HbA1c (%)	9.3	5.6	—	—	4–6

**FIGURE 1:**
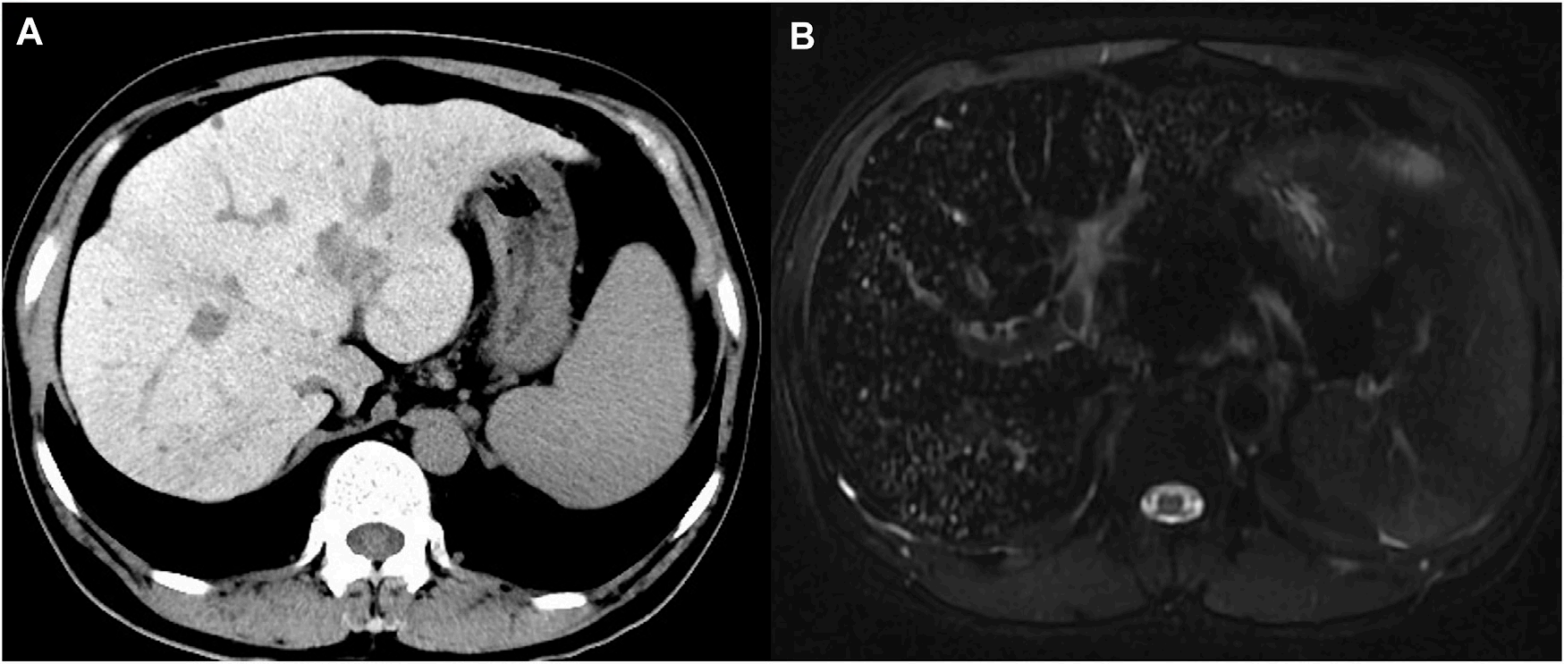
Computed tomography and magnetic resonance imaging of the proband. **(A)** Precontrast CT showed hyper-attenuation of liver parenchyma; **(B)** MRI T2-weighted image showed diffuse low signal intensity of liver parenchyma obviously.

Pre-contrast computed tomography (CT) revealed hepatomegaly with an increased attenuation over the liver and spleen, splenomegaly, and collateral circulation formation (Figure 1A). Magnetic resonance imaging (MRI) showed a typical decrease in T2-weighted signal intensity over the liver (Figure 1B).

**FIGURE 2:**
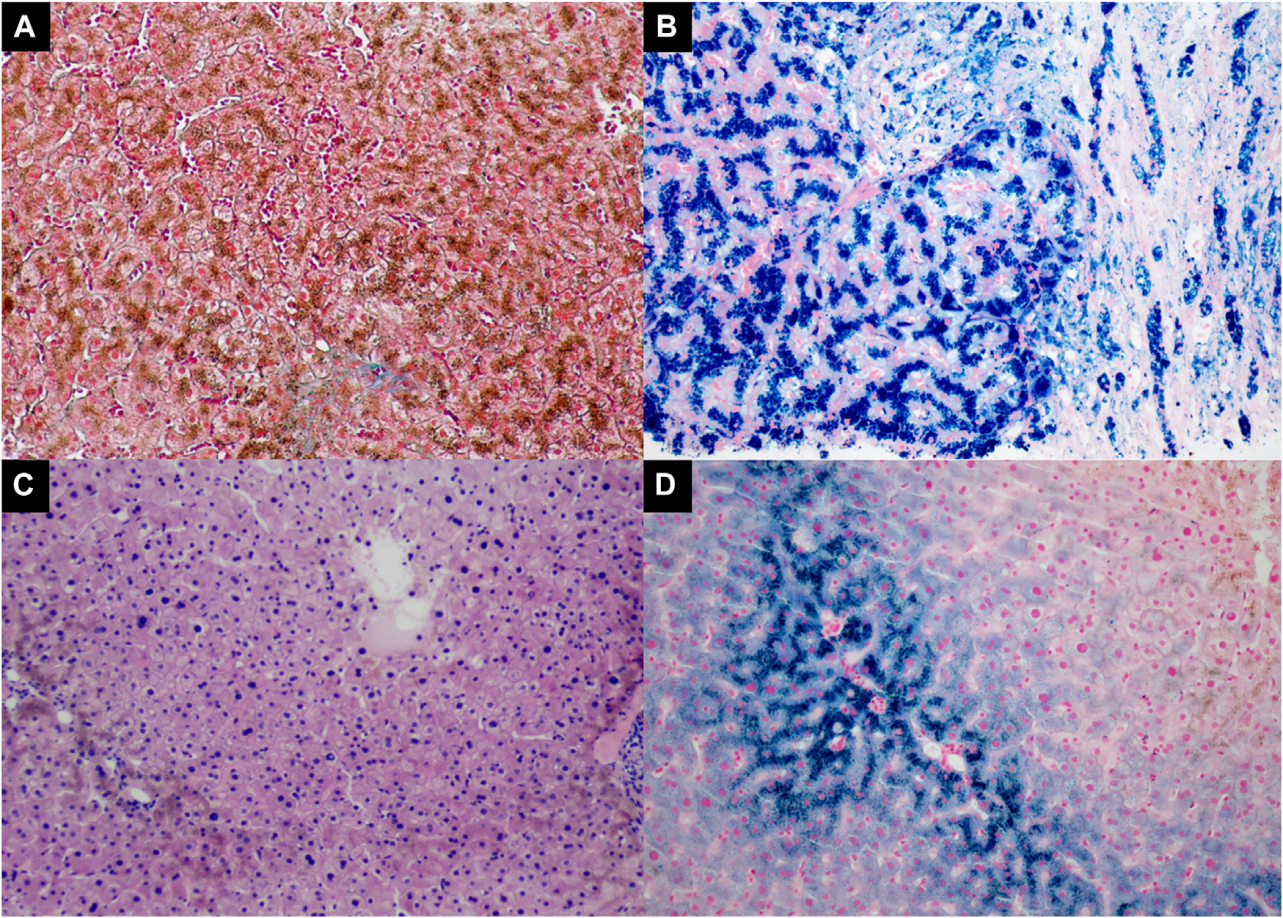
The liver histopathology of the proband [Panel **(A, B)**, II-1] and the proband’s sister [Panel **(C, D)**, II-3]. **(A)** Increased collagen fibers stained green by Masson Trichrome stain in the expanded fibrous portal tract with cirrhosis (Masson trichrome, 100×); **(B)** Marked iron deposition in the hepatocytes and biliary epithelium (Prussian blue, 200×); **(C)** Marked deposition of hemosiderin in the hepatocytes (H&E, 100×); **(D)** The proband’s sister had iron deposited in the periportal hepatocytes (Prussian blue, 200×).

A liver biopsy was performed, and the liver histology was evaluated by an experienced pathologist. The findings confirmed established cirrhosis with a marked deposition of iron in the hepatocytes and biliary epithelia, which is consistent with hemochromatosis (Figures 2A,B).

We also investigated one of the patient’s sisters (Figure 4 *,* II-3), and the results showed that she had abnormal plasma iron indices as well: iron, 37 μmol/L; transferrin saturation, 94.9%; and ferritin>709 ng/ml (Table 1), while her HbA1c and sex hormones levels were normal (HbA1c 5.6%, estradiol <37 pmol/L, follicle stimulating hormone 58.02 IU/L, luteinizing hormone 25.86 IU/L, progesterone 0.5 nmol/L, prolactin 464.13 mIU/L, and testosterone 0.94 nmol/L). Also, a fine needle aspiration biopsy was performed, which showed iron deposition in the periportal hepatocytes (Figures 2C,D).

### Genetic Testing Results

Genetic analysis was performed for the patient and his family to explore the genetic factor for hemochromatosis. Panel sequencing was performed for major genes involved in the liver metabolism and carbohydrate metabolism including the common HH-related geness, namely, *HFE*, *HJV*, *HAMP*, *TFR2*, and *SLC40A1*. The SureSelect Human All ExonV6 custom chip was used to capture exons, and the Illumina sequencing platform was used for high-throughput sequencing. BWA (0.7.12-r1039) (Li and Durbin, 2009) was used to compare next-generation sequencing data with the human genome; ANNOVAR ($ Date: 2015-06-17) (Park and Park, 2021) was used to annotate mutation sites according to dbSNP, Clinvar, ExAC, 1,000 Genomes, and other databases. Possible pathogenicity of gene mutation was graded by the ACMG (American Society of Medical Genetics and Genomics) genetic mutation grading system. Then, the target sequence of the suspected pathogenic mutation was amplified by PCR and sequenced *via* an ABI3730xL sequencer (Applied Biosystems, Foster City, CA, United States). Finally, the comparisons and analysis for Sanger sequencing data were performed by SeqMan (DNASTAR, Madison, Wisconsin, United States). Potential functional effects of detected missense mutations were predicted by PolyPhen-2 (http://genetics.bwh.harvard.edu/pph2/index.shtml), SIFT (http://sift.jcvi.org/), PROVEAN (http://provean.jcvi.org/index.php), MutationTaster (http://mutationtaster.org/), and FATHMM (http://fathmm.biocompute.org.uk/).

The results showed that the patient (II-1) had new heterozygous mutations c.1288G > A (p.G430R)/c.960T > A (p.Y320X) in the *TFR2* gene. These mutations were further confirmed by Sanger sequencing (Supplementary Figure S1). As a result, the novel c.960T > A mutation led to a premature stop codon at amino acid 320 (p.Y320X). According to the ACMG guideline, the mutation c.960T > A (p.Y320X) was identified as pathogenic (PVS1: null variant + PM2: absent from controls + PP4: the phenotype of the patient is highly consistent with HH type 3). The mutation c.1288G > A (p.G430R) is a pathogenic mutation that has been reported earlier (Majore et al., 2013). Here, we also predicted its pathogenicity by utilizing six kinds of prediction software. All software programs except for FATHMM showed that the p.G430R mutation was damaging (Table 2). The mutation was identified as likely pathogenic according to the ACMG guideline (PM1: the variant is in the domain + PM3: there is a literature suggesting this variant + PM2: absent from controls + PP3: bioinformatics protein function comprehensive prediction software predicts outcomes as harmful + PP4: the phenotype of the patient is highly consistent with HH type 3). The PhastCons scores of these two mutations were both 1, and the corresponding PhyloP values were 4.594 and 4.844, respectively, suggesting the high conservation of these two amino acids. Moreover, amino acid sequence alignments for the corresponding parts in the TFR2 protein in multiple species were studied (Supplementary Figure S3). The results showed that the two amino acids at positions 430 and 320 were absolutely conserved.

**TABLE 2:** Bioinformatics analysis of the *TFR2* mutations.

Mutation	PolyPhen2		SIFT		PROVEAN		MutationTaster		FATHMM		ACMG
	Score	Prediction	Score	Prediction	Score	Prediction	Score	Prediction	Score	Prediction	Prediction
c.1288G > A	1	Probably	0	Deleterious	−6.81	Deleterious	1	Disease causing	0.51	Tolerated	Likely pathogenic
p.G430R		damaging									
c.960T > A	—	—	—	—	—	—	1	Disease causing	—	—	Pathogenic
p.Y320X											

**FIGURE 3:**
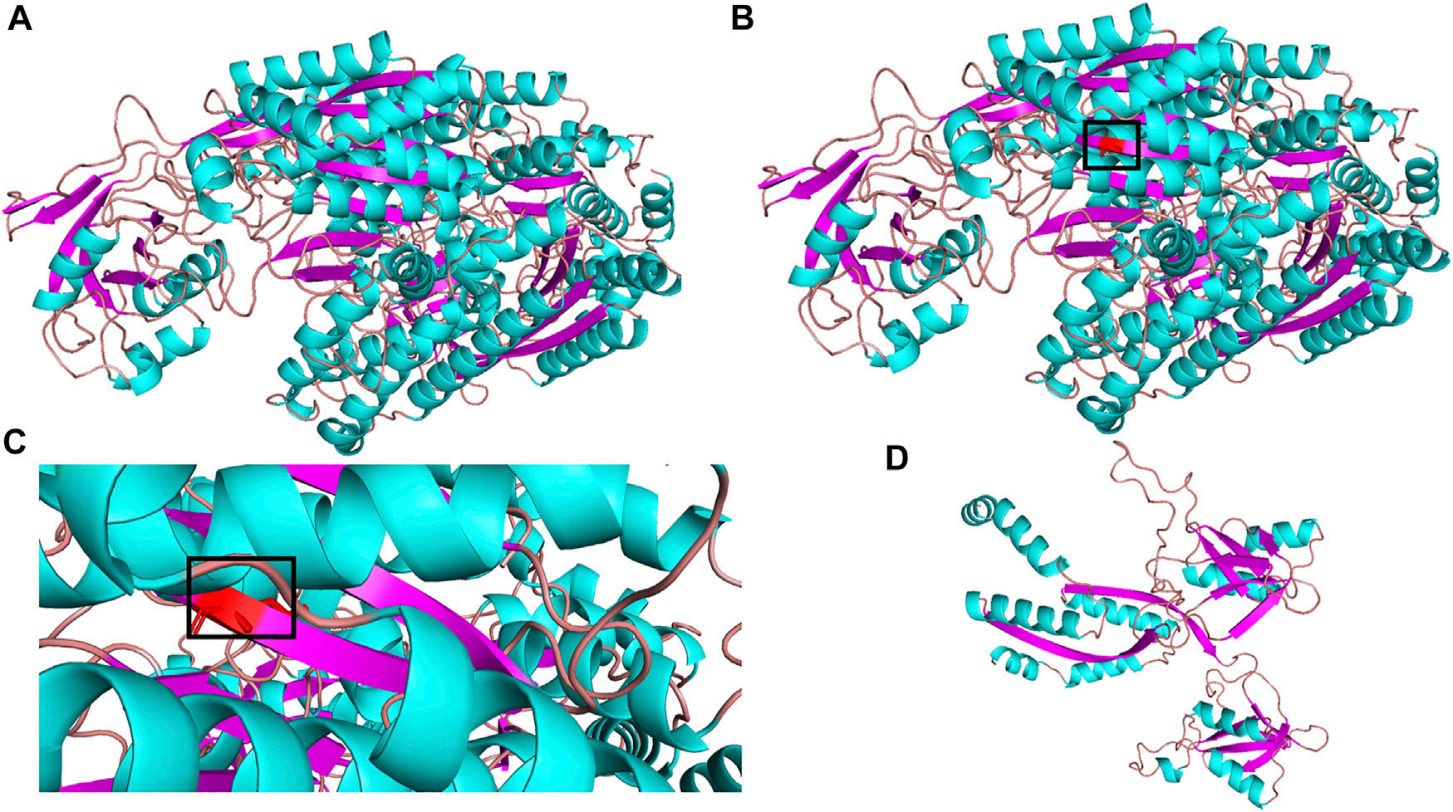
3D structure of TFR2 protein. **(A)** wild-type; **(B)** mutant type c.G1288A (p.G430R); **(C)** partial mutation graph c.G1288A (p.G430R); **(D)** mutant type c.T960A (p.Y320X).

The molecular structure of *TFR2* was constructed by SWISS-MODEL (https://swissmodel.expasy.org/interactive). Also, molecular graphics were analyzed by PyMOL software. As shown in Figure 3 *,* the secondary structure of the predicted mutation was not affected by the mutation c.1288G > A (p.G430R) but affected by the mutation c.960T > A (p.Y320X). There is no electricity in the blank space in the model evaluation graph (Supplementary Figure S2), indicating that the model quality is acceptable.

**FIGURE 4:**
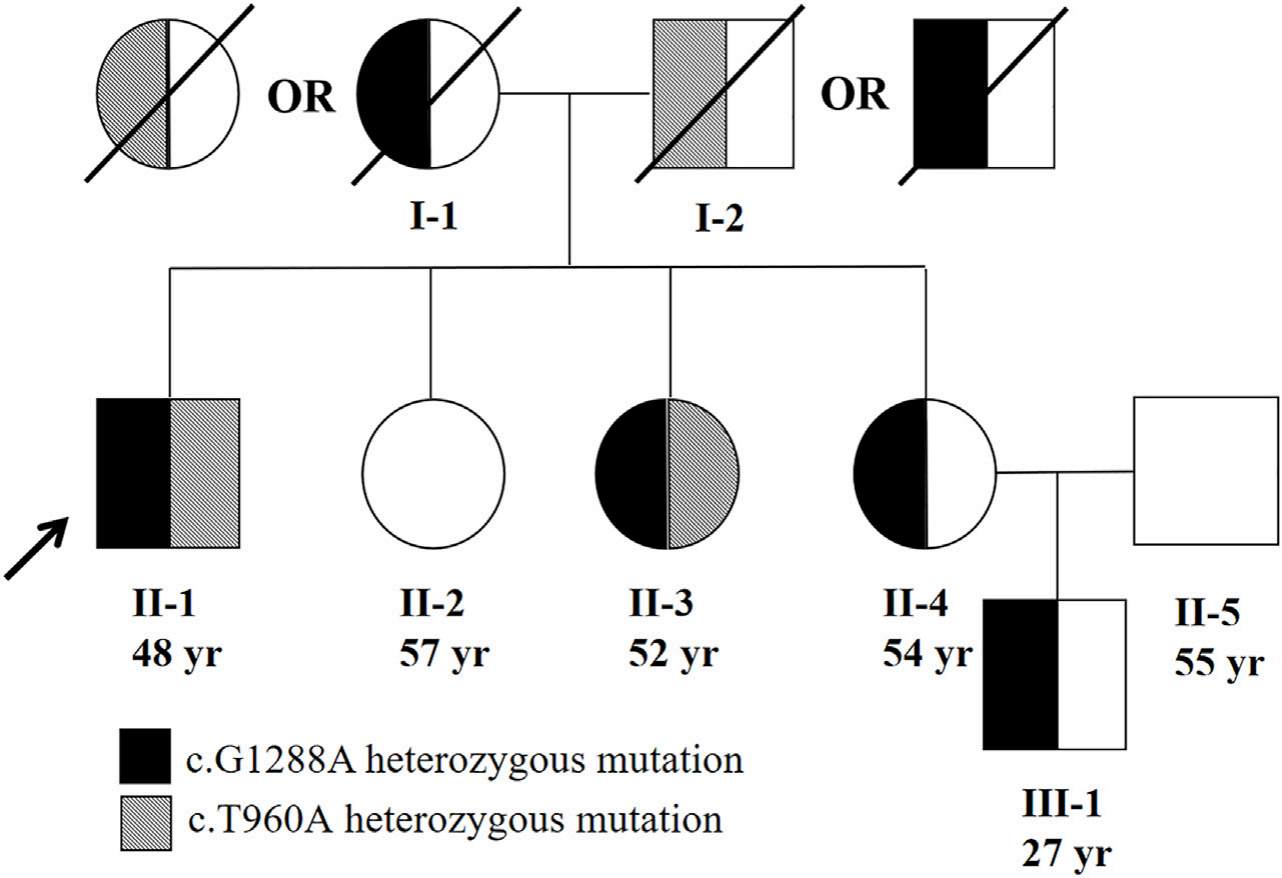
Family pedigree of patient. The parents of the proband should each had a heterozygous mutation (c.960T > A or c.G1288A). The proband’s parents were not consanguineous.

Moreover, we tested the mutations in the *TFR2* gene for his family members (Figure 4). The same heterozygous mutations were found in one of his sisters (II-3). In addition, one of the patient’s sisters (II-4) was found to carry a heterozygous mutation c.1288G > A (p.G430R). However, *TFR2* gene mutation was not found in the other sister (II-2). The patient’s parents had passed away, so we cannot perform their genetic sequencing. According to the genetic sequencing results of his sisters, we speculated the mutations in the proband were compound heterozygous. In addition, the nephew (III-1) of the proband was also confirmed to have a heterozygous mutation c.1288G > A (p.G430R).

## Ethical Statement

All procedures involving human participants were in accordance with the ethical standards of the Institute Ethical Committee of Beijing YouAn Hospital, Capital Medical University, Beijing, China, and with the Helsinki Declaration (as revised in 2013). Written informed consent was obtained from the patient and his family members.

## Discussion

In recent years, increasing mutations in the *TFR2* gene which are related to type 3 HH have been documented (Hernández et al., 2021; Joshi et al., 2015). As shown in Supplementary Table S1, a total of 33 pathogenic *TFR2* mutations associated with HH have been described in the Human Gene Mutation Database (Stenson et al., 2020). Most patients were Caucasian (27/33, 81.8%), males (17/25, 68.0%). The average age of the patients was 31.4 years, and most of them had liver cirrhosis. The data on the mutations in the *TFR2* gene in Asian patients are relatively rare with only five articles reported.

In the present study, we described a Chinese Han pedigree suffering from type 3 HH with a novel compound heterozygous mutation c.1288G > A (p.G430R)/c.960T > A (p.Y320X) in the *TFR2* gene. The proband exhibits very high plasma transferrin saturation and deposition of iron in the hepatocytes and biliary epithelia, and this phenotype highly suggested hemochromatosis. The gene sequencing results confirm the diagnosis of type 3 hemochromatosis. One of his sisters shares the same *TFR2* gene mutation; however, her liver pathology is milder than that in the proband. Except for the hepatic manifestations, other clinical features including diabetes, hypogonadism, and skin pigmentation have been reported in patients with type 3 hemochromatosis. Given this, we evaluated these symptoms in the proband and his family members. As a result, the proband developed skin pigmentation and diabetes, while his sister showed no other extrahepatic manifestations. We considered that the difference in the phenotype between the proband and his sister can be ascribed to other factors including sex (menstruation), environment (iron intake), or possibly other unknown genetic factors. These factors apart from *TFR2* gene mutation probably contribute to the development of HH. At present, both the proband and his sister have received phlebotomy therapy and resulted in improvement in the laboratory test (Table 1).

This case report highlights three important clinical issues. First, our team discovered the *TFR2* gene c.960T > A (p.Y320X) mutation, which led to a premature stop codon at amino acid 320 (p.Y320X). According to the ACMG genetic mutation grading system, this mutation is pathogenic. We also analyzed the predictive mutation structure and found that the Y320X mutation affects the secondary structure of the *TFR2* protein and may destroy its function. Our report expands the mutation spectrum of the *TFR2* gene associated with HH. Second, this is the first time to report a novel compound heterozygous mutation c. G1288A (p.G430R)/c.T960A (p.Y320X) in the *TFR2* gene in a Chinese pedigree. We analyzed the minor allele frequency (MAF) of the c. G1288A (p.G430R) mutation by utilizing different public databases in different populations (Supplementary Table S2). The MAF of c. G1288A (p.G430R) is between 0.000003978 and 0.000008 in different databases, which indicates that the mutation is extremely rare. Third, early diagnosis and treatment for patients with HH have been documented to improve the prognosis as well as decrease mortality. It is helpful to remind even physicians in Asia that where the incidence of HH is low, it is important to keep the diagnosis of HH in mind, when a patient is presented with symptoms such as iron overload, liver fibrosis, diabetes, and so on.

## CONCLUSION

In conclusion, we first reported a Chinese pedigree of *TFR2*-related hemochromatosis with a novel compound heterozygous mutation c.1288G > A (p.G430R)/c.960T > A (p.Y320X). Our report broadens the mutation spectrum of the *TFR2* gene associated with HH. On the other hand, our results reveal inconsistency between genotypes and phenotypes, which indicates multifactorial etiology contributing to the development of HH.

## Data Availability

The original contributions presented in the study are included in the article/Supplementary Material, further inquiries can be directed to the corresponding authors.
