# Supplementary material for: A Novel Mutation of Transferrin Receptor 2 in a Chinese Pedigree With Type 3 Hemochromatosis: A Case Report
Source: Front Genet. 2022 Apr 8;13:836431. doi: 10.3389/fgene.2022.836431 (PMC9024051; doi:10.3389/fgene.2022.836431)
Supplement: Supplementary file 1 [file DataSheet1.docx]

**Supplementary table 1. The information on HH patients with mutations in the TFR2 gene**

| **Number** | **Ancestry** | **Exon** | **Codon change** | **Amino acid change** | **Sex** | **Age**  **(yr)** | **Phenotypes** | | | | **Reference** |
| --- | --- | --- | --- | --- | --- | --- | --- | --- | --- | --- | --- |
|  |  |  |  |  |  |  | **liver cirrhosis** | **diabetes** | **Pigmentation** | **Other*** |  |
| 1 | Italian | 2 | 64G > A | V22I | - | - | - | - | - | - | ^[1]^ |
| 2 | North French | 2 | 313C>G | R105T | F | 15 | - | - | - | - | ^[2]^ |
| 3 | Italian | 3 | 381C>A | D127E | - | - | - | - | - | - | ^[3]^ |
| 4 | Italian | 4 | 515T>A | M172K | M | 43 | Yes | No | No | Yes | ^[4]^ |
| 5 | Italian | 6 | 750C>G | Y250X | - | - | - | - | - | - | ^[5]^ |
| 6 | Spanish | 6 | 840C>G | F280L | M | 28 | - | - | - | - | ^[6]^ |
| 7 | Spanish | 7 | 916C>T | Q306X | M | 12 | - | - | - | - | ^[7]^ |
| 8 | Italian | 7 | 949C>T | Q317X | M | 21 | Yes | No | Yes | No | ^[8]^ |
| 9 | Spanish | 9 | 1117G>A | G373D | F | 59 | - | - | - | - | ^[9]^ |
| 10 | Italian | 9 | 1128C>A | A376D | - | - | - | - | - | - | ^[3]^ |
| 11 | American | 10 | 1186C>T | R396X | M | 48 | Yes | No | Yes | Yes | ^[10]^ |
| 12 | Japanese | 10 | 1206C>A | N402K | F | 79 | Yes | Yes | Yes | No | ^[11]^ |
| 13 | French | 11 | 1235A>T | N412I | M | 17 | No | Yes | No | No | ^[12]^ |
| 14 | Spanish | 11 | 1259G>A | R420H | M | 37 | - | - | - | - | ^[13]^ |
| 15 | African | 11 | 1288G>A | G430R | M | 49 | Yes | No | No | No | ^[14]^ |
| 16 | French | 12 | 1330G>A | A444T | F | 21 | - | - | - | - | ^[15]^ |
| 17 | Asiatic | 12 | 1364G>A | R455Q | - | - | - | - | - | - | ^[16]^ |
| 18 | Chinese | 13 | 1403G>A | R468H | - | - | - | - | - | - | ^[17]^ |
| 19 | Mexican | 13 | 1409G>T | S470I | F | 2 | - | - | - | - | ^[18]^ |
| 20 | Japanese | 13 | 1469T>G | L490R | F | 49 | Yes | Yes | Yes | No | ^[19]^ |
| 21 | African | 13 | 1511A>G | Y504C | M | 49 | Yes | No | No | No | ^[14]^ |
| 22 | Spanish | 15 | 2014C>T | Q672X | M | 25 | - | - | - | - | ^[20]^ |
| 23 | French | 15 | 2033G>C | R678P | F | 10 | No | No | No | No | ^[12]^ |
| 24 | Asiatic | 15 | 2038G > T | D680Y | M | 35 | - | - | - | - | ^[20]^ |
| 25 | Portuguese | 15 | 2069A>C | Q690P | M | 29 | Yes | No | Yes | Yes | ^[21]^ |
| 26 | Italian | 15 | 2176G>A | D726N | - | - | - | - | - | - | ^[3]^ |
| 27 | French | 15 | 2188C>T | R730C | F | 29 | Yes | No | Yes | Yes | ^[12]^ |
| 28 | French | 15 | 2203G>A | G735S | M | 25 | No | No | Yes | No | ^[12]^ |
| 29 | Italian | 15 | 2219C>T | T740M | M | 28 | - | - | - | - | ^[22]^ |
| 30 | Portuguese | 15 | 2249T>C | L750P | - | - | - | - | - | - | ^[23]^ |
| 31 | Portuguese | 15 | 2330C>T | A777V | M | 36 | - | - | - | - | ^[23]^ |
| 32 | Spanish | 15 | 2343G>A | W781X | M | 41 | - | - | - | - | ^[20]^ |
| 33 | American | 15 | 2374G>A | G792R | M | 48 | Yes | No | Yes | Yes | ^[10]^ |

Note:F, female; M, male; -, not mentioned; Yes, presence; No, absence; * Hypogonadism, cardiovascular disease and other clinical manifestations

**Supplementary table 2. The minor allele frequency (MAF) in different populations**

| Mutation | 1000 Genomes Project | | | | Exome Aggregation Consortium | | | | Genome Aggregation Database | | | |
| --- | --- | --- | --- | --- | --- | --- | --- | --- | --- | --- | --- | --- |
|  | All | East Asian | South Asian | caucasian | All | East Asian | South Asian | caucasian | All | East Asian | South Asian | caucasian |
| c.1288G>A  p.G430R | - | - | - | - | 0.000008 | 0 | 0 | 0.00001 | 0.000003978 | 0 | 0.00003266 | 0 |
| c.960T>A  p.Y320X | - | - | - | - | - | - | - | - | - | - | - | - |

Note: -,unannotated


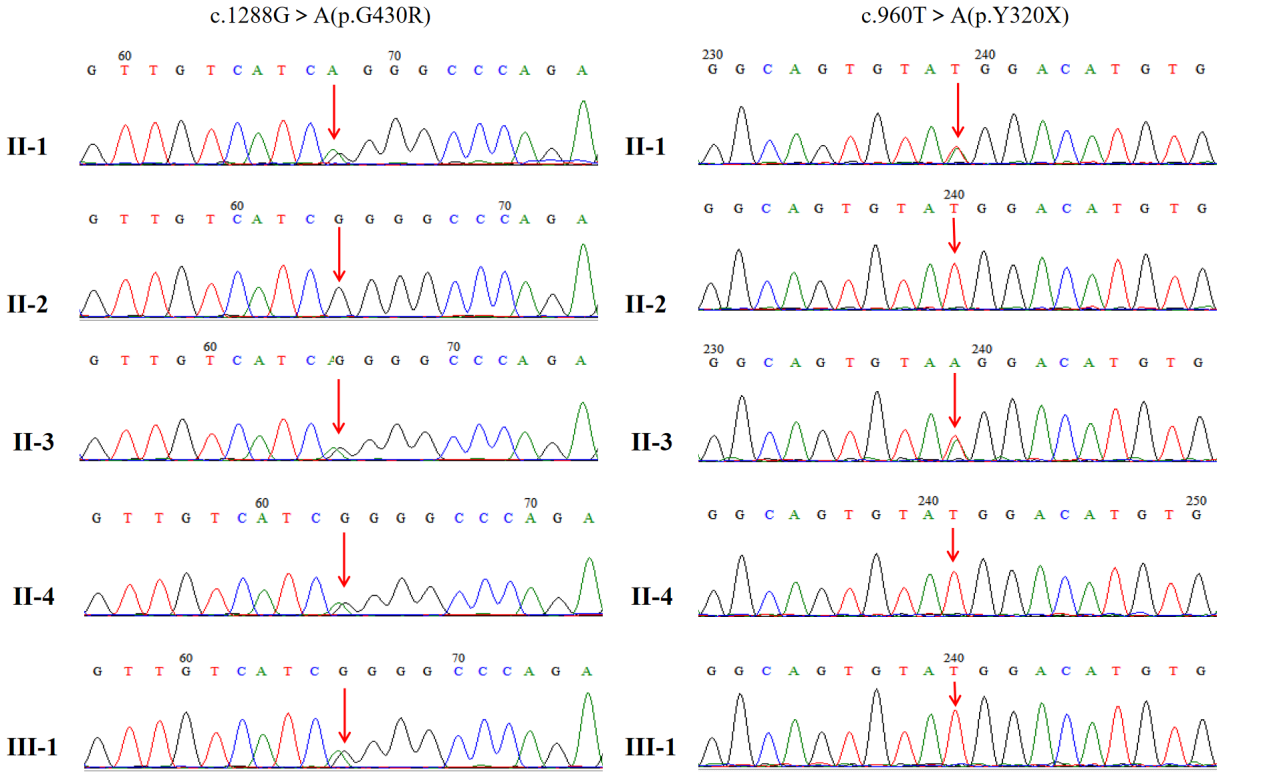


Figure S1. Sanger sequencing reveals two heterozygous mutations in *TFR2* gene, c.1288G＞A(p.G430R) and c.960T＞A (p.Y320X)


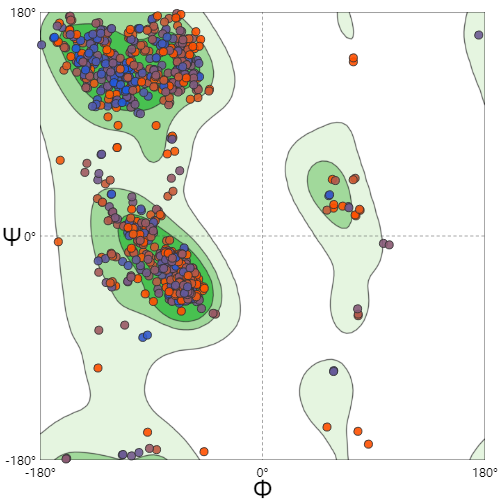


Figure S2. The 3D structure model evaluation graph for TFR2 protein


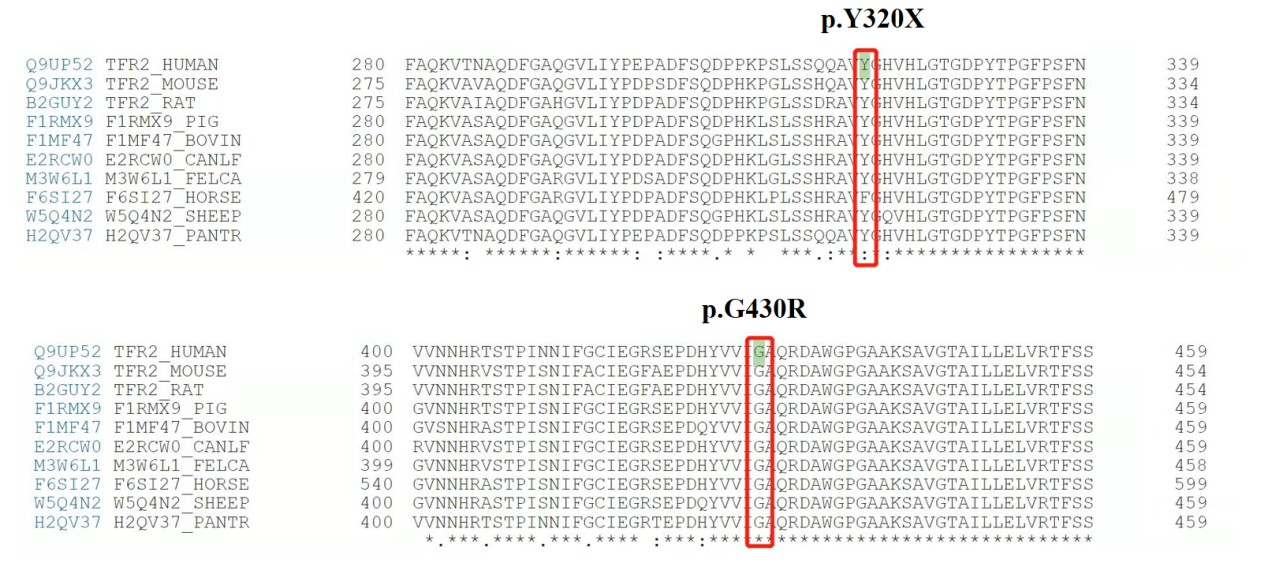


Figure S3. Partial amino acid sequence alignment of TFR2 protein in 10 animal species with p.G430R and p.Y320X mutations. Uniprot accession numbers are reported for each sequence. Below the alignment a star (*) indicates 100% conservation of the amino acid, semicolons and dots indicate amino acids with similar but not identical properties.

**Reference**

[1] Biasiotto G, Belloli S, Ruggeri G, et al. Identification of new mutations of the HFE, hepcidin, and transferrin receptor 2 genes by denaturing HPLC analysis of individuals with biochemical indications of iron overload[J]. Clin Chem, 2003, 49(12): 1981-1988.

[2] Le Gac G, Mons F, Jacolot S, et al. Early onset hereditary hemochromatosis resulting from a novel TFR2 gene nonsense mutation (R105X) in two siblings of north French descent[J]. Br J Haematol, 2004, 125(5): 674-678.

[3] Badar S, Busti F, Ferrarini A, et al. Identification of novel mutations in hemochromatosis genes by targeted next generation sequencing in Italian patients with unexplained iron overload[J]. Am J Hematol, 2016, 91(4): 420-425.

[4] Roetto A, Totaro A, Piperno A, et al. New mutations inactivating transferrin receptor 2 in hemochromatosis type 3[J]. Blood, 2001, 97(9): 2555-2560.

[5] Aguilar-Martinez P, Esculie-Coste C, Bismuth M, et al. Transferrin receptor-2 gene and non-C282Y homozygous patients with hemochromatosis[J]. Blood Cells Mol Dis, 2001, 27(1): 290-293.

[6] Mendes A I, Ferro A, Martins R, et al. Non-classical hereditary hemochromatosis in Portugal: novel mutations identified in iron metabolism-related genes[J]. Ann Hematol, 2009, 88(3): 229-234.

[7] Joshi R, Shvartsman M, Moran E, et al. Functional consequences of transferrin receptor-2 mutations causing hereditary hemochromatosis type 3[J]. Mol Genet Genomic Med, 2015, 3(3): 221-232.

[8] Pietrangelo A, Caleffi A, Henrion J, et al. Juvenile hemochromatosis associated with pathogenic mutations of adult hemochromatosis genes[J]. Gastroenterology, 2005, 128(2): 470-479.

[9] Del-Castillo-Rueda A, Moreno-Carralero M I, Cuadrado-Grande N, et al. Mutations in the HFE, TFR2, and SLC40A1 genes in patients with hemochromatosis[J]. Gene, 2012, 508(1): 15-20.

[10] Lee P L, Barton J C. Hemochromatosis and severe iron overload associated with compound heterozygosity for TFR2 R455Q and two novel mutations TFR2 R396X and G792R[J]. Acta Haematol, 2006, 115(1-2): 102-105.

[11] Yamashita T, Morotomi N, Sohda T, et al. A male patient with ferroportin disease B and a female patient with iron overload similar to ferroportin disease B[J]. Clin J Gastroenterol, 2014, 7(3): 260-264.

[12] Bardou-Jacquet E, Cunat S, Beaumont-Epinette M P, et al. Variable age of onset and clinical severity in transferrin receptor 2 related haemochromatosis: novel observations[J]. Br J Haematol, 2013, 162(2): 278-281.

[13] Del C A, Moreno-Carralero M I, Cuadrado-Grande N, et al. [Hyperferritinemia, ferropenia and metabolic syndrome in a patient with a new mutation of gene TFR2 and another in gene FTL. A family study][J]. Med Clin (Barc), 2011, 137(2): 68-72.

[14] Majore S, Ricerca B M, Radio F C, et al. Type 3 hereditary hemochromatosis in a patient from sub-Saharan Africa: is there a link between African iron overload and TFR2 dysfunction?[J]. Blood Cells Mol Dis, 2013, 50(1): 31-32.

[15] Biasiotto G, Camaschella C, Forni G L, et al. New TFR2 mutations in young Italian patients with hemochromatosis[J]. Haematologica, 2008, 93(2): 309-310.

[16] Hofmann W K, Tong X J, Ajioka R S, et al. Mutation analysis of transferrin-receptor 2 in patients with atypical hemochromatosis[J]. Blood, 2002, 100(3): 1099-1100.

[17] Hsiao P J, Tsai K B, Shin S J, et al. A novel mutation of transferrin receptor 2 in a Taiwanese woman with type 3 hemochromatosis[J]. J Hepatol, 2007, 47(2): 303-306.

[18] Khayat A A, Suchi M, Vitola B. A Rare Case Of a 2-year-old Boy With Alagille Syndrome and Type 3 Hereditary Hemochromatosis With TFR2 Mutation[J]. J Pediatr Gastroenterol Nutr, 2019, 68(4): e68-e70.

[19] Koyama C, Wakusawa S, Hayashi H, et al. Two novel mutations, L490R and V561X, of the transferrin receptor 2 gene in Japanese patients with hemochromatosis[J]. Haematologica, 2005, 90(3): 302-307.

[20] Hernandez G, Ferrer-Cortes X, Venturi V, et al. New Mutations in HFE2 and TFR2 Genes Causing Non HFE-Related Hereditary Hemochromatosis[J]. Genes (Basel), 2021, 12(12).

[21] Mattman A, Huntsman D, Lockitch G, et al. Transferrin receptor 2 (TfR2) and HFE mutational analysis in non-C282Y iron overload: identification of a novel TfR2 mutation[J]. Blood, 2002, 100(3): 1075-1077.

[22] Radio F C, Majore S, Binni F, et al. TFR2-related hereditary hemochromatosis as a frequent cause of primary iron overload in patients from Central-Southern Italy[J]. Blood Cells Mol Dis, 2014, 52(2-3): 83-87.

[23] Faria R, Silva B, Silva C, et al. Next-generation sequencing of hereditary hemochromatosis-related genes: Novel likely pathogenic variants found in the Portuguese population[J]. Blood Cells Mol Dis, 2016, 61: 10-15.
